# Beyond Comparing Machine Learning and Logistic Regression in Clinical Prediction Modelling: Shifting from Model Debate to Data Quality

**DOI:** 10.2196/77721

**Published:** 2025-11-05

**Authors:** Yanan Hu, Xin Zhang, Valerie Slavin, Yitayeh Belsti, Sofonyas Abebaw Tiruneh, Emily Callander, Joanne Enticott

**Affiliations:** 1 Monash Centre for Health Research and Implementation Faculty of Medicine, Nursing and Health Sciences Monash University Melbourne Australia; 2 Department of Electrical and Computer Systems Engineering Monash University Melbourne Australia; 3 Gold Coast Hospital and Health Service Gold Coast Hospital Gold Coast Australia; 4 School of Nursing and Midwifery Griffith University Gold Coast Australia; 5 School of Nursing and Midwifery University of Technology Sydney Sydney Australia; 6 School of Nursing, Midwifery and Social Work The University of Queensland Brisbane Australia; 7 School of Public Health University of Technology Sydney Sydney Australia

**Keywords:** machine learning, clinical prediction models, logistic regression, clinical data, data quality, binary classfication

## Abstract

The rapid uptake of supervised machine learning (ML) in clinical prediction modelling, particularly for binary outcomes based on tabular data, has sparked debate about its comparative advantage over traditional statistical logistic regression. Although ML has demonstrated superiority in unstructured data domains, its performance gains in structured, tabular clinical datasets remain inconsistent and context dependent. This viewpoint synthesizes recent comparative studies and simulation findings to argue that there is no universal best modelling approach. Model performance depends heavily on dataset characteristics (eg, linearity, sample size, number of candidate predictors, minority class proportion) and data quality (eg, completeness, accuracy). Consequently, we argue that efforts to improve data quality, not model complexity, are more likely to enhance the reliability and real-world utility of clinical prediction models.

## Introduction

The increasing adoption of supervised machine learning (ML) in clinical prediction models (diagnostic or prognostic) based on tabular data has sparked considerable debate regarding its comparative performance against traditional statistical logistic regression (LR), particularly for binary outcomes such as mortality or the occurrence of adverse events [1]. Although supervised ML approaches have demonstrated clear superiority in classifying unstructured clinical data such as medical images and texts [2], their added value for the classification of clinical tabular data, where structured data are organized in tables, typically with rows representing individual cases and columns representing individual characteristics, remains uncertain and context-dependent [3].

## Definition of Statistical LR Versus ML-Based LR

The distinction between statistical LR and ML-based LR is frequently blurred in both literature and practice [1]. Many studies loosely refer to any penalized LR model as ML, despite fundamental methodological differences. To enable valid comparisons between these approaches, it is therefore crucial to clearly delineate their boundaries (Table 1).

**Table 1:** Definitions of statistical logistic regression and machine learning–based logistic regression.

Method	Definition
Statistical logistic regression	A parametric model operating under conventional statistical assumptions, including linearity and independence, employing fixed hyperparameters without data-driven optimization, using prespecified candidate predictors based on clinical or theoretical justification. This aligns with traditional epidemiological approaches where model specification precedes data analysis.
Machine learning–based logistic regression	An adaptive variant where model specification becomes part of the analytical process itself, hyperparameters like penalty terms are tuned through cross-validation, predictors may be selected algorithmically from a broader set of candidates, and the analytical focus shifts decisively toward predictive performance. While mathematically similar to statistical logistic regression, this approach embodies the machine learning philosophy of learning from data.

In this paper, we adopt the definition proposed in a previous systematic review [1], which characterizes statistical LR as a theory-based model that operates under strict assumptions and does not involve data-driven optimization of predictive performance through hyperparameter tuning but relies on subject knowledge from researchers or experts to specify the model structure. Although penalized LR models may involve hyperparameter tuning and variable selection, they remain theory-involved and do not intrinsically capture nonlinearities or interactions, and the core assumptions of LR still apply. Conversely, ML models are defined as methods that autonomously learn patterns from data (ie, data-driven hyperparameter tuning or predictor selection) [4], including ML-based LR and other supervised ML methods (eg, random forest, boosting, neural networks, support vector machine) that intrinsically handle complex interactions without manual specification beforehand.

## Comparative Performance: What Was Already Known

A 2019 meta-regression of 145 low-risk-of-bias comparisons between statistical LR and ML binary clinical prediction models on tabular data showed no performance benefit of ML over statistical LR [1]. However, this comparison was limited to discrimination measured by the area under the receiver operating characteristic curve (AUROC), as other performance metrics were not frequently reported. Notably, 79% (56/71) of the studies did not report calibration performance, and only one study reported clinical utility. Clinical utility is commonly assessed through decision curve analysis, which estimates the clinical value of a prediction model at the population level by considering the consequences of decisions made based on its output [5], specifically, the benefit of correctly predicting true positives and the harm of incorrectly predicting false positives. A step-by-step guide to this method is available here [6].

Each performance metric captures a distinct aspect of model performance, with its own strengths and limitations. A model may achieve a high AUROC yet still have poor calibration and potentially harmful clinical consequences if the predicted probabilities are systematically overestimated or underestimated; vice versa, a well-calibrated model may still have poor discriminative ability. This highlights the need for comprehensive evaluation across multiple performance domains, including discrimination, calibration, classification metrics, clinical utility, and fairness. Therefore, focusing solely on marginal gains in AUROC between LR and ML can be misleading and inadequate for guiding future research.

Furthermore, the meta-regression of AUROC did not explore the underlying sources of performance differences (eg, sample size, number of predictors, use of hyperparameter tuning). Therefore, it is still unclear whether the observed variation in performance reflects true algorithmic superiority or is instead driven by dataset characteristics or modelling procedures. For example, the systematic review highlighted that more than half of the included studies did not clearly report their hyperparameter tuning strategies.

The comparison should not only focus on the difference in performance but also on the stability of performance [7]. Even when statistical LR outperforms ML in certain metrics (eg, AUROC), this does not necessarily imply that the predictions are stable or reproducible, that is, applying the same model development procedure to different samples of the same size drawn from the same underlying population can result in substantially different predictions for the same individual. This issue is particularly pronounced when using small development datasets, which leads to more different models in the multiverse, often with vastly unstable individual predictions [8]. Adherence to the minimum sample size recommendations is one way to mitigate this issue [9,10]. Notably, a 2023 systematic review reported that 73% of the binary clinical prediction models using statistical LR had sample sizes below the recommended minimum threshold [11]. ML algorithms are generally more data-hungry than LR to achieve stable performance. For example, one study demonstrated that random forest may require more than 20 times the number of events for each candidate predictor compared to statistical LR [10].

## Research Recommendation for Reporting: Improving the Transparency of Each Step

In light of the methodological issues identified in the current literature rather than focusing solely on determining the inherent superiority of one modelling approach over another, greater attention should be directed toward ensuring the rigor and transparency of modelling procedures. This includes clear documentation of data preprocessing steps, sample size justifications, modelling decisions, hyperparameter tuning strategies (eg, grid or random search), feature selection techniques (including filter methods like correlation analysis, wrapper methods like recursive feature elimination, or embedded methods such as least absolute shrinkage and selection operator [LASSO]), model performance evaluation methods and metrics, and model explanation methods (eg, Shapley Additive Explanations [SHAP] [12], Submodular Pick Local Interpretable Model-agnostic Explanations [SP-LIME] [13], and Counterfactual Explanations for Robustness, Transparency, Interpretability, and Fairness of Artificial Intelligence [CERTIFAI] models [14]).

## No Free Lunch Theorem

There is no universal golden method for clinical prediction models [15,16] and whether the benefit of each algorithm (either statistical or ML) can be fulfilled is highly subject to the dataset characteristics (eg, sample size, class imbalance, nonlinearity, number of candidate predictors [10]) and data quality (eg, completeness, accuracy [17]).

Each algorithm has its unique strengths and limitations in handling different data characteristics (Table 2). For example, Categorical Boosting is particularly effective for datasets with high-cardinality categorical variables, as it includes built-in techniques to encode categories without extensive preprocessing [18]. eXtreme Gradient Boosting [19] and Light Gradient-Boosting Machine [20] are known for their computational efficiency, performance in data with complex feature interactions, and native handling of missing data, but are less interpretable than LR. Deep learning, a subfield of ML, uses multilayered neural networks to simulate human decision-making [21]. While capable of learning highly complex nonlinear relationships from extremely large and high-dimensional datasets, deep learning models are generally more data-hungry, less interpretable, and require significantly more computational resources than traditional ML methods, which may limit their transparency and clinical applicability [22]. Although efforts in Explainable Artificial Intelligence (XAI) are advancing, current ML models often fall short of the level of clarity and trust required for clinical implementation [23]. On the other hand, LR is highly interpretable and performs well on small sample sizes when predictors have an approximately linear relationship with the outcome, but it may struggle with complex nonlinearities or large numbers of correlated predictors. The smaller the sample size available, the more we must rely on external information or inputs from experts to determine the features/predictors.

**Table 2:** Strengths and weaknesses of statistical logistic regression and machine learning in binary clinical prediction models based on tabular data.

Aspect	Statistical logistic regression	Supervised machine learning
Learning process	Theory-driven; relies on expert knowledge for model specification and candidate predictor selection	Data-driven; directly and automatically learn relationship from data
Assumptions in data structure	High (eg, interactions, linearity)	Low; handle complex, nonlinear relationship
Assumptions in model specification	High; use default value	Low; data-driven hyperparameter tuning
User input in creation and selection of candidate predictors	High; researchers need to investigate the nonlinearity of continuous variables and interaction effects and systematic review or expert opinion of candidate predictors before developing the model	Low; models automatically capture nonlinearity and interactions, no need for researchers to investigate nonlinearity and interaction effects between variables
Flexibility	Low; constrained by linearity assumptions but can be improved by adding penalty	High
Complexity	Low; simple, parametric model	High
Performance on complex data	Low	High
Sample size requirement for stable performance	Low	High; data-hungriness
Interpretability (in-processing decision-making process)	High; white-box nature, model coefficients are directly interpretable, can also be presented using graphical score charts or nomograms	Low; black-box nature, decision-making process is not transparent
Explainability (postprocessing explanation)	High	Low; complex to explain to end users, requires post hoc methods like Shapley Additive Explanations for explanation
Deployment ease	High	Low
Computational cost	Low	High

Therefore, the choice of the algorithm should be tailored to the structure, quality, and characteristics of the dataset. Ultimately, the development of clinical prediction models involves unavoidable trade-offs. There is no single algorithm that excels across all performance metrics (fairness, accuracy, generalizability, stability, parsimony, and interpretability). Researchers must prioritize certain metrics depending on the model’s intended application and target population. For instance, model parsimony, where a model with fewer predictors may sacrifice some accuracy for simplicity, can be crucial in enhancing user acceptance, as overly complex models may reduce usability. Additionally, discussions with stakeholders (eg, health care providers, patients) regarding the most relevant features or desired trade-offs can guide model development.

Clinical tabular datasets often exhibit characteristics that tend to favor LR over ML models [9]. These include small to moderate sample sizes, relatively high levels of noise, a limited number of candidate predictors (ie, low dimension), and typically binary outcomes (Table 3). Such conditions can constrain the ability of complex ML algorithms to demonstrate superior performance. Moreover, LR’s well-recognized interpretability and trustworthiness [24] further reinforce its widespread use in clinical prediction modelling, and it is typically used as a reference model for performance benchmarking in ML studies. However, ML approaches may warrant consideration when they demonstrate clear superiority in performance, supported by model explainability to help build trust among clinicians and end users. To date, no consensus exists on how to evaluate or compare model interpretability and explainability across different methods [23].

**Table 3:** Mismatches between the characteristics of clinical data and supervised machine learning’s strengths.

Aspect	Characteristics in clinical data	Supervised machine learning’s relative strength compared to statistical logistic regression	Comments
Data modality	Mostly single modal data (tabular data)	Excels with multimodal data (image, scan, text, or signal)	Clinical datasets often lack the multimodal richness that enables ML^a^ models to fully demonstrate their advantages
Data quality	High noise due to errors, missingness, or inconsistent measurement (low signal-to-noise ratio)	Performs better for data with high signal-to-noise ratio	Noise dilutes true signals, and ML models tend to overfit to noisy artifacts without careful data preprocessing
Sample size	Often small to moderate	Benefit from large-scale datasets. More “data-hungry”	Although sample sizes are improving in some registries, they are often insufficient to train complex ML architectures robustly
Predictors	Typically, a small set of clinically meaningful predictors with high linearity and low order of interaction terms	Excels with high-dimensional, nonlinear interactions, temporally rich data	ML's strength in handling high-dimensional, time-series data with higher nonlinearity and interaction terms
Prediction	Predominantly binary classification (eg, event occurrence: yes/no).	Advantage in multiclass classification and regression	Simple binary classification problems often diminish the additional complexity that ML can handle

^a^ML: machine learning.

## Policy and Research Recommendation: Shifting From Model Debate to Data Quality

Amid increasing interest in complex models, it is crucial to reorient clinical prediction modelling from a model-centric to a data-centric paradigm [25]. The quality, structure, and representativeness of data are far more critical to model performance than the complexity of models. In clinical settings, prediction models serve best as a second set of eyes, complementing clinical judgment rather than replacing it [23]. However, without high-quality data, even the most sophisticated models will propagate existing biases and limitations, as the saying goes, “garbage in, garbage out.”

As the number of clinical prediction models continues to grow, policymakers and funding bodies should prioritize investment in data quality infrastructure, including standardized phenotyping, consistent variable definitions, and robust data curation practices. Since all models are trained on historical data that inherently reflect systemic limitations, model complexity cannot resolve errors rooted in the data; in fact, they might amplify the bias and make unfair decisions in underrepresented or marginal groups such as defined by sex, ethnicity, or deprivation [26]. In contrast, thoughtful data preprocessing and transparent reporting of modelling strategies are foundational to developing reliable, generalizable, reproducible, and trustworthy decision support tools [27]. In addition, more effort is needed to expand the candidate predictors available in health data [26], such as integrating lifestyle factors collected through wearables and a range of medical devices [28].

This shift in emphasis from modelling sophistication to data stewardship is essential to ensure that clinical prediction tools genuinely enhance, rather than undermine, the quality and equity of patient-centered care.

## Abbreviations

AUROC: area under the receiver operating characteristic curve
CERTIFAI: Counterfactual Explanations for Robustness, Transparency, Interpretability, and Fairness of Artificial Intelligence
LASSO: least absolute shrinkage and selection operator
LR: logistic regression
ML: machine learning
SHAP: Shapley Additive Explanations
SP-LIME: Submodular Pick Local Interpretable Model-agnostic Explanations
XAI: Explainable Artificial Intelligence
